# From cancer therapy to cardiac safety: the role of proteostasis in drug-induced cardiotoxicity

**DOI:** 10.3389/fphar.2024.1472387

**Published:** 2024-11-14

**Authors:** Xingyu Qian, Mengdong Yao, Jingyu Xu, Nianguo Dong, Si Chen

**Affiliations:** ^1^ Department of Cardiovascular Surgery, Union Hospital, Tongji Medical College, Huazhong University of Science and Technology, Wuhan, Hubei, China; ^2^ Cancer Center, Union Hospital, Tongji Medical College, Huazhong University of Science and Technology, Wuhan, Hubei, China; ^3^ Department and Institute of Urology, Tongji Hospital, Tongji Medical College, Huazhong University of Science and Technology, Wuhan, Hubei, China

**Keywords:** drug-induced cardiotoxicity, protein unfolding, protein misfolding, chemical denaturation, endoplasmic reticulum stress

## Abstract

Drug-induced cardiotoxicity (DICT) poses a significant challenge in the prognosis of cancer patients, particularly with the use of antineoplastic agents like anthracyclines and targeted therapies such as trastuzumab. This review delves into the intricate interplay between drugs and proteins within cardiac cells, focusing on the role of proteostasis as a therapeutic target for mitigating cardiotoxicity. We explore the *in vivo* modeling of proteostasis, highlighting the complex intracellular environment and the emerging techniques for monitoring proteostasis. Additionally, we discuss how cardiotoxic drugs disrupt protein homeostasis through direct chemical denaturation, endoplasmic reticulum stress, unfolded protein response, chaperone dysfunction, impairment of the proteasome system, and dysregulation of autophagy. Finally, we provide insights into the applications of cardioprotective drugs targeting proteostasis to prevent cardiotoxicity and the adoption of structural proteomics to evaluate potential cardiotoxicity. By gaining a deeper understanding of the role of proteostasis underlying DICT, we can pave the way for the development of targeted therapeutic strategies to safeguard cardiac function while maximizing the therapeutic potential of antineoplastic drugs.

## 1 Introduction

Drug-induced Cardiotoxicity (DICT) has become a major challenge limiting drug application scenarios, most typically with antineoplastic drugs (Barachini et al., 2023). Among them, anthracyclines were first noted to induce cardiotoxicity manifested as progressive heart failure (Cardinale et al., 2020). While the introduction of more precisely targeted drugs was initially intended to lead to a better prognosis, some unanticipated cardiovascular adverse events have certainly cast a dark shadow. In parallel with the benefits of trastuzumab, which targets epidermal growth factor receptor-2 (HER2), 27% of patients experience cardiac dysfunction, and 16% experience symptomatic heart failure (Slamon et al., 2001). Extensive reviews of breast cancer survivors indicated a greater risk of death from cardiovascular disease, exceeding their initial risk of death from cancer itself, especially in older patients (Chen et al., 2012; Siegel et al., 2012). In addition to this, immune checkpoint inhibitors have been shown to be associated with inflammation-related cardiovascular adverse events, particularly myocarditis (odds ratio 11.21, 95% CI 9.36–13.43) (Salem et al., 2018; Varricchi et al., 2018b). Statistically, up to 27 drugs have been forced off the market in the last few decades due to severe cardiotoxicity (Mamoshina et al., 2021). Hence, elucidating the mechanisms of DICT is of great significance for the broader application of existing agents and the development of new drugs. The plethora of unsolved issues has even spawned the emerging discipline of cardio-oncology (Bellinger et al., 2015).

Various mechanisms concerning DICT have been elucidated, of which mitochondrial dysfunction (Rocca et al., 2022), activation of apoptotic signaling (Christidi and Brunham, 2021), and calcium channel dysfunction (Herrmann, 2020) have been widely recognized. Several reviews have already summarized the mechanisms of DICT, establishing a bridge from microscopic reactions to macroscopic effects and explaining how specific mechanisms of cardiotoxic drugs lead to cardiotoxicity (Herrmann, 2020; Morelli et al., 2022; Abdul-Rahman et al., 2023) (Table 1).

**TABLE 1:** Main cardiotoxicity and mechanisms of antineoplastic drugs.

Drug	Main cardiotoxicity	Mechanism	Ref
Doxorubicin	Cardiac rhythm changes, altering blood pressure, pericarditis, myocarditis, cardiomyopathy, and congestive heart failure	Promoting ROS generation, thereby triggering the caspase-3 apoptotic pathway; promoting ROS generation, thereby inducing lysosome acidification and leading to autophagy	Dickey and Rao (2012), Ghigo et al. (2016), Ma et al. (2020), Sheibani et al. (2022), Barachini et al. (2023), Li et al. (2024a)
Tyrosine kinase inhibitors (afatinib, ponatinib, and sorafenib)	Congestive heart failure, myocardial ischemia, hypertension,peripheral artery disease (10%) and myocardial infarction, arterial occlusion, arrhythmia	Inducing transient ROS followed by lipid peroxidation, causing cardiac damages	Fu et al. (2016), Wang et al. (2023)
Bevacizumab	Reduced left ventricular ejection fraction, hypertension, and arrhythmia	Leading to mitochondrial dysfunction of cardiomyocytes, reducing ATP production, increasing ROS generation, attenuated antioxidant enzyme levels, initiating the apoptosis	Van Leeuwen et al. (2020), Huang et al. (2021), Yetkin-Arik et al. (2021)
Cisplatin	Electrocardiographic changes, myocarditis, arrhythmia, congestive heart failure, and cardiomyopathy	Inducing cardiotoxicity by modulating inflammatory and endoplasmic reticulum stress responses	Al-Majed et al. (2006), Chowdhury et al. (2016)
5-Fluorouracil	Arrhythmias, myocardial infarction and sudden cardiac death	5-FU activates the endoplasmic reticulum stress ATF6 pathway, increases the expression of GRP78 and ATF6, affects the function of cardiomyocytes, and induces cardiotoxicity	Sorrentino et al. (2012), Wang et al. (2024)
Celastrol	QT prolongation, cardiomyocyte apoptosis and cardiac injury	Causing oxidative stress responses *in vivo*, activating the TNF signaling pathway and the caspase family, and leading to cell apoptosis	Sun et al. (2006), Liu et al. (2019)
HSP70/90 inhibitor	hERG trafficking inhibition, long QT syndrome, ventricular arrhythmias	Promoting the generation of ROS and stimulating ER stress, ROS-mediated activation of CaMKII promotes the activation of calcium channels, triggering hERG trafficking inhibition	Ficker et al. (2003)
Proteasome inhibitor (Carfilzomib, Bortezomib)	Hypertension, pulmonary hypertension, heart failure, arrhythmias, ischaemic heart diseases, and thromboembolism	Promoting the generation of inflammatory cytokines and apoptotic markers, such as IL-1β, IL-6, TNFα, and caspase-3	Wu et al. (2020), Zheng et al. (2023)

Behind these mechanisms, unexpected drug-protein interactions play a crucial role. Protein homeostasis, abbreviated as proteostasis, is the dynamic equilibrium of protein concentration, localization, and conformation within any living cell. Dependence on molecular chaperones, proteolytic machinery, endoplasmic reticulum (ER), and their regulators enables protein synthesis, folding, and degradation to occur under scrutiny (Hoppe and Cohen, 2020). However, under the stress of changing metabolic, environmental, and pathological conditions, unfolded or unfolded proteins accumulate, thus constantly jeopardizing proteostasis (Hipp et al., 2019). A growing body of evidence indicates that diseases such as Alzheimer’s and diabetes are associated with the accumulation of misfolded proteins (Cozachenco et al., 2023). Among the numerous identified risk factors, pharmaceutical agents, particularly antineoplastic drugs, present a significant challenge to proteostasis. Tumor cells rely on a high degree of proteostasis to cope with their genetic instability and the tumor microenvironment, and a considerable number of antineoplastic drugs target this feature (Liang et al., 2023). However, once off-target effects arise, they can lead to irreversible adverse consequences for relatively vulnerable normal cells. Therefore, resolving the alterations in the conformation and stability of proteins in cardiac cells (cardiomyocytes, endothelial cells, immune cells, etc.) induced by drugs is essential for targeting and rescuing cardiotoxicity and expanding the scope of application of existing drugs (Wang et al., 2020).

Evidence from *in vitro* experiments demonstrates that various denaturants, such as heat, chemicals, pressure, and force, cause distinct unfolding processes (Lapidus, 2017). Similarly, pharmaceuticals can induce the unfolding process by functioning as chemical denaturants *in vivo* (Ayaz et al., 2023). In addition to this, endoplasmic reticulum stress, molecular chaperone dysfunction, proteasome dysfunction, and impaired autophagy pathways together contribute to perturbed proteostasis in the heart. By reviewing recent advances in the study of drug-induced cardiotoxicity, we aimed to provide a novel insight into the drug-protein interaction *in vivo*. Together with the evaluation of *in vivo* monitoring techniques and targeted interventions, we explored the potential of proteostasis in preventing DICT.

## 2 *In vivo* modeling of proteostasis

Current computational techniques that rely on sequence similarity have been quite effective in predicting the native structures of proteins (Jeppesen and André, 2023). However, these methods do not adequately tackle the task of identifying folding intermediates. This situation is regrettable since comprehending the folding and unfolding routes of specific proteins is crucial for our understanding of many proteostasis disorders (Sari et al., 2024). The initial research into the mechanism of polypeptide chain folding during protein synthesis was primarily concerned with thermodynamic aspects, such as the calculation of folding free energy. Nobel laureate B. F. Anfinsen postulated in his work on the spontaneous refolding of bovine pancreatic ribonuclease—a protein with a disulfide bond—that denatured proteins could be refolded *in vitro* in the 1960s (Vila, 2023). Typically, *in vitro* tests involve treating the protein with substances like urea, guanidine hydrochloride, or heat to unfold, and then removing these substances to allow the protein to regain its original structure (Seelig and Seelig, 2023). In addition to X-rays, nuclear magnetic resonance (NMR) (Zhuravleva and Korzhnev, 2017) and circular dichroism (CD) spectroscopy (Singh et al., 2015), techniques that tend to provide structural information, advanced experimental techniques, such as single-molecule fluorescence resonance energy transfer (FRET), may effectively determine the duration of transition path times and therefore provide insights into the timing of important folding processes (Barth et al., 2019).

**FIGURE 1:**
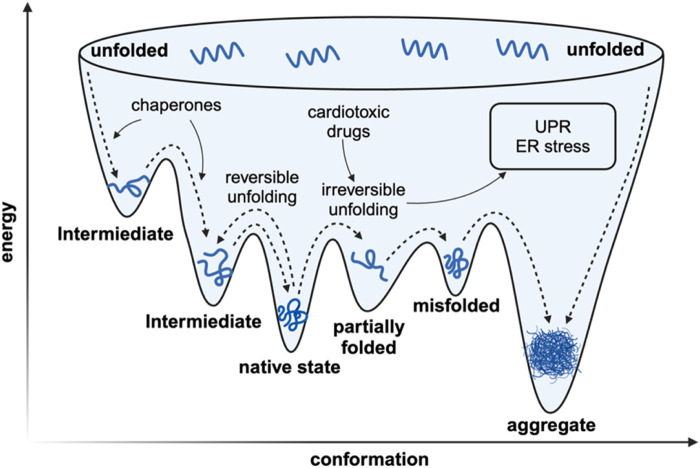
The simplified landscape of proteostasis *in vivo*. The *in vivo* rapid folding pathway begins with the creation of a high-energy state by the ribosome/chaperone complex. The protein’s inherent structure is located at a flexible local thermodynamic minimum. If the protein deviates from its local thermodynamic minimum, it can have two outcomes: it can transition to another local minimum and undergo reversible denaturation, or it can adopt a conformation with significantly lower Gibbs free energy, which prevents it from refolding and leads to irreversible denaturation characterized with pathogenic aggregation.

As for living organisms, a sophisticated network of molecular processes is engaged to ensure the integrity and functionality of the proteome (Figure 1). Protein biogenesis is achieved through the recognition of nascent polypeptide chains within the ribosomal tunnels and the utilization of molecular chaperones to ensure the accurate folding of newly translated polypeptides (Burke et al., 2022). The endoplasmic reticulum is the site where post-translational modification and protein folding occur. Folding abnormalities of nascent peptides in ER due to the interference of endogenous and exogenous factors will cause the accumulation of unfolded and misfolded proteins, which will ultimately cause ER stress (Oakes and Papa, 2015). Once misfolded proteins are further accumulated, the unfolded protein response (UPR) will be activated to reduce ER burden by enhancing the endoplasmic reticulum-associated protein degradation (ERAD), thus maintaining proteostasis (Senft and Ronai, 2015). Fully processed proteins are then transported to their respective organelles, where they engage in specific enzymatic or structural functions. In the event of protein damage or the necessity for protein recycling, these proteins are degraded through two main processes: the ubiquitin-proteasome system and autophagy. Parts of the misfolded proteins can be refolded by specialized molecular chaperones or, if not, eventually degraded by the proteasome. While the other portion of misfolded protein proteins that are prone to aggregation accumulate to form toxic oligomers and even pathogenic aggregates (Pohl and Dikic, 2019).

Significant progress has been achieved in recent years in the visualization and monitoring of intracellular proteostasis. In essence, these methods can be classified into four principal categories: optical-based techniques, mass spectrometry-based techniques, intracellular nuclear magnetic resonance (NMR) techniques and cryo-electron microscopy techniques (Bai et al., 2023). The observation of oligomers and protein aggregates can be achieved through the utilization of specific fluorescent probes, such as Tetraphenylethene Maleimide (TPE-MI) developed by (Tang et al., 2021). Mass spectrometry-based methodologies offer a distinct advantage in the identification of individual protein species, as they permit the visualization of the protein structure (de Souza and Picotti, 2020). Moreover, intracellular NMR techniques afford a comprehensive insight into the dynamic behavior of a single protein, both prior to and following a misfolding event (Luchinat et al., 2022). The rapid evolution of cryo-electron tomography techniques demonstrates considerable potential for visualizing the structure and properties of protein aggregates within complex and crowded intracellular environments (Watanabe et al., 2020). The aforementioned technical tools afford researchers an intuitive view of how drugs affect proteostasis.

## 3 Cardiotoxic drugs disrupt proteostasis

Drugs usually bind to certain proteins *in vivo*, changing the target protein’s and downstream proteins' activities and, eventually, stopping the biological process that caused the illness (Galluzzi et al., 2024). Inhibition of the same target protein in the heart, though, can have a major adverse effect (Kitakata et al., 2022). Among these, the buildup of unfolded or misfolded proteins caused by drugs poses a major obstacle to comprehending and treating cardiac dysfunction.

Urea and guanidine chloride (GdmCl) have been widely utilized as chemical denaturants in both research and industrial facilities for an extended period of time (Paladino et al., 2023). A urea molecule forms five interactions with the protein moiety, while a Gdm + ion forms six interactions with the protein moiety and has a notable affinity for aromatic side chains (Paladino et al., 2022). Their denaturing impact is based on their heterophilicity. The primary factor that causes unfolding is the rise in entropy resulting from the generation of a vast number of conformational microstates due to the occupation and non-occupation of the binding site (Seelig and Seelig, 2023). Interestingly, urea-based anticancer agents have grown by leaps and bounds over the past few decades (Listro et al., 2022), especially a series of protein kinase inhibitors including sorafenib (Wilhelm et al., 2006) and regorafenib (Dhillon, 2018). The urea moiety is supposed to be introduced to serve as a favorable scaffold and to enhance affinity with the substrate (Ghosh and Brindisi, 2020), but such drugs also have the ability to act as *in vivo* denaturants, and therefore tight blood concentration monitoring is necessary.

**FIGURE 2:**
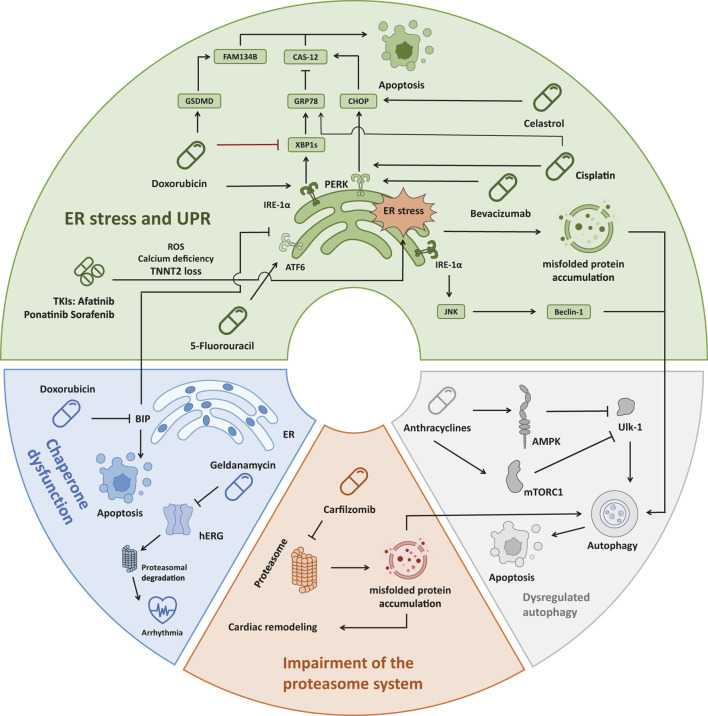
Four main mechanisms of drug-induced accumulation of unfolded/misfolded proteins leading to cardiac dysfunction. (1) ER stress: Doxorubicin activates IRE-1α, which leads to JNK/Beclin-1 pathway activation and autophagy. Doxorubicin also inhibited the expression of XBP1s, which prevented the induction of sufficient GRP78, leading to the activation of Caspase-12 and cell apoptosis. Doxorubicin also induced the cleavage and activation of GSDMD, causing ER stress, and activated FAM134B to promote cell apoptosis. Three TKIs (Afatinib, Ponatinib, Sorafenib) caused ER stress through ROS, Calcium deficiency, and TNNT2 loss, resulting in cardiotoxicity. Bevacizumab, Cisplatin, and Celastrol all promote the PERK-CHOP pathway to induce apoptosis. In addition, Cisplatin could also affect GRP78 in the XBP1s-GRP78 pathway to initiate apoptosis. 5-Fluorouracil triggers cardiotoxicity by interacting with the ATF6 pathway. (2) Chaperone dysfunction: Doxorubicin reduced the expression of the chaperone protein BIP in the ER, leading to cell apoptosis. The chaperone inhibitor Geldanamycin irreversibly causes misfolding, increases proteasomal degradation of hERG and triggers arrhythmia. (3) Impairment of the proteasome system: Carfilzomib caused proteasome inhibition in 80% of patients, resulting in misfolded protein accumulation in cardiomyocytes and aggravation of cardiac remodeling. (4) Dysregulated autophagy: Anthracyclines inhibit Ulk-1 through AMPK and mTORC1, thereby weakening the protective autophagy pathway and inducing apoptosis. ER, endoplasmic reticulum; IRE1α, inositol-requiring protein 1α; JNK, c-Jun N-terminal kinase; XBP1, X-box-binding protein 1; GRP78, glucose-regulated protein 78; GSDMD, gasdermin; TKI, tyrosine kinase inhibitor; ROS, reactive oxygen species; TNNT2, human cardiac troponin T gene; PERK, protein kinase RNA-like ER kinase; CHOP, C/EBP homologous protein; ATF6, activating transcription factor 6; Ulk-1, unc-51-like autophagy activating kinase 1; AMPK, AMP-activated protein kinase; mTOR, mammalian target of Rapamycin.

In addition to the direct action of denaturants, there are multiple mechanisms that lead to disequilibrium in proteostasis, including ER stress, unfolded protein response (UPR), chaperone dysfunction, impairment of the proteasome system, and dysregulation of autophagy (Henning and Brundel, 2017; Ren et al., 2021). ER stress and UPR, initiated by the accumulation of misfolded proteins within the ER, trigger a cascade of signaling events aimed at restoring protein homeostasis but can ultimately induce apoptosis and lead to cardiac dysfunction if unresolved (Foufelle and Fromenty, 2016). Uncontrolled ER stress and UPR induce apoptosis by a variety of pathways, such as the promotion of C/EBP homologous protein (CHOP) expression by activating transcription factor 4 (ATF4), inhibiting the B cell lymphoma protein 2 (BCL-2) protein family to induce apoptosis (Ren et al., 2021). Inositol-requiring enzyme-1 (IRE-1) can also form a complex with tumor necrosis factor receptor-associated factor-2 (TRAF2) and apoptosis signal-regulating kinase-1 (ASK1), activating c-Jun N-terminal kinase (JNK) signaling as well as caspase-12 (Sano and Reed, 2013). The activating transcription factor 6α (ATF6α) can also induce apoptosis by regulating CHOP expression or downregulating myeloid cell leukemia-1 (MCL-1) (Adachi et al., 2008; Fernández et al., 2015). Chaperones play a crucial role in assisting protein folding and preventing aggregation. Meanwhile, Hsp 70, a major category of chaperons, can directly interact with apoptotic protease-activating factor 1 (Apaf-1) which is a key regulator of the caspase-dependent apoptotic pathway and prevents oligomerization of Apaf-1 with procaspase-9, thereby inhibiting apoptosome formation (Ravagnan et al., 2001). However, the expression and function of the chaperones can be compromised by drug-induced stressors, which can lead to uncontrolled ER stress and apoptosis (Mayer and Bukau, 2005; Fu et al., 2016). Additionally, impairment of the proteasome system, responsible for degrading misfolded proteins, further exacerbates protein accumulation within cardiac cells (Li et al., 2020; Zheng et al., 2023). These abnormal ubiquitinated protein aggregates can induce cellular injury and ultimately lead to caspase-mediated apoptosis and cell death (Hasinoff et al., 2017). Moreover, dysregulation of autophagy, a vital cellular process for degrading protein aggregates, can contribute to the accumulation of toxic protein species and subsequent cardiac dysfunction (Kim et al., 2011; Nazarko and Zhong, 2013; Li et al., 2020) (Figure 2).

Understanding the intricate interplay between these pathways is essential for developing targeted therapeutic strategies to alleviate drug-induced cardiac dysfunction (Table 2).

**TABLE 2:** Drugs inducing cardiotoxicity through disrupting proteostasis.

Drug	Pathway	Model	Ref
Doxorubicin	ER stress and UPR	mice model, *in vivo*	Fu et al. (2016), Kong et al. (2022), Qu et al. (2022)
	Chaperone dysfunction	mice model, *in vivo*	Fu et al. (2016)
	Dysregulation of autophagy	mice model, *in vivo*	Li et al. (2018)
Tyrosine kinase inhibitors (afatinib, ponatinib, and sorafenib)	ER stress and UPR	Human induced pluripotent stem cell-derived cardiomyocytes and RNAseq analysis	Fu et al. (2016), Wang et al. (2023)
Bevacizumab	ER stress and UPR	H9C2 cell lines,*in vitro*	Li et al. (2021)
Cisplatin	ER stress and UPR	mice model, *in vivo*	Chowdhury et al. (2016)
5-Fluorouracil	ER stress and UPR	mice model, *in vivo* and vitro	Wang et al. (2024)
Celastrol	ER stress and UPR	mice model, *in vivo*	Chen et al. (2022)
HSP70/90 inhibitor	Chaperone dysfunction	The HEK-hERG13 (HEK 293 stably transfected with human hERG WT) cell line, *in vitro*	Peterson et al. (2012)
Carfilzomib	Proteasome inhibition	case reports	Grandin et al. (2015)

### 3.1 ER stress and UPR

About one-third of the human proteome is synthesized in ER, especially secreted proteins and membrane proteins, which play an important role in the maintenance of intracellular protein homeostasis (Díaz-Villanueva et al., 2015; Schwarz and Blower, 2016). Folding enzymes and chaperons in the ER lumen assist in the correct folding of polypeptides. For instance, the protein disulfide isomerase (PDI) family, which contains 20 specific enzymes, catalyzes the formation of disulfide bonds. Chaperone proteins perform quality control on protein folding, such as BiP, an HSP70 that recognizes hydrophobic residues in unfolded proteins, collaborating with DnaJ (Hsp40) to promote the refolding of the unfolded proteins by continuous circuit of binding and releasing unfolded proteins (Halperin et al., 2014).

Although there are various mechanisms to ensure the correct folding of proteins in the ER, once the increasing demand for protein synthesis leads to the overload of the ER folding protein beyond its capacity, or adverse events damage the function of the ER folding protein, abnormal state of the ER function rises, which is called ER stress. The significant feature of ER stress is the accumulation of unfolded or misfolded proteins (Díaz-Villanueva et al., 2015). There are various mechanisms to eliminate the unfolded or misfolded proteins, the most important of which is the UPR. UPR has three branches, respectively starting from inositol-requiring protein 1α (IRE1α), activating transcription factor 6 (ATF6), and protein kinase RNA-like ER kinase (PERK) (Hetz et al., 2020). They are both ER-resident transmembrane proteins, whose lumen parts bind to BiP to be inhibited in an inactive state (Ren et al., 2021), when there are abundant unfolded proteins in the ER, BiP is more inclined to bind to the unfolded proteins, thereby freeing IRE1α, ATF6, and PERK to active states and triggering UPR. After BiP dissociation, IRE1α and PERK spontaneously form homodimers. Homodimerized IRE1α activates endoribonuclease activity to catalyze the splicing of the mRNA encoding the transcription factor X-box-binding protein 1 (XBP1) (Jurkin et al., 2014). XBP1 transcription factor promotes transcription and translation of genes involved in the synthesis of ER chaperone proteins and folding enzymes, thereby enhancing ER protein folding capacity (Shen et al., 2001; Yoshida et al., 2001; Calfon et al., 2002). The IRE1α/XBP1 pathway is essential for the activation of ER-associated degradation (ERAD), promoting the degradation of unfolded/misfolded protein (Yoshida et al., 2003). Activated PERK promotes dimerization and self-phosphorylation of eukaryotic translation initiation factor 2A (eIF2A), reducing the synthesis of various proteins, which alleviates the unfolded/misfolded protein load (Zielke et al., 2021). However, the aforementioned process promotes the expression of ATF4, which regulates the transcription of genes involved in protein synthesis and folding, amino acid metabolism, and redox control, reducing the accumulation of unfolded and misfolded proteins in the ER (Fusakio et al., 2016). For the ATF pathway, the dissociation of BiP promotes the transfer of ATF molecules to the Golgi apparatus, and the site-1 and site-2 proteases in the Golgi apparatus hydrolyze luminal and transmembrane domains of ATF6 respectively. Activated ATF6 interacts with ER stress response elements 74 and regulates the expression of genes encoding the components XBP1, BiP, and ERAD (Jin et al., 2017; Hetz et al., 2020; Ren et al., 2021). Generally, the unfolded/misfolded protein accumulation be eliminated by the above mechanisms, if not, decompensation of ER stress will lead to functional damage of cells, and in the most severe cases, to apoptosis.

The accumulation of unfolded or misfolded proteins can participate in the functional damage of cardiomyocytes through ER stress, which is both a cause and a consequence (Ren et al., 2021). Varieties of cardiotoxic drugs (Bagchi et al., 2021; Chen et al., 2022; Wang et al., 2023) can induce ER stress of cardiomyocytes, in turn, aggravating cardiac dysfunction.

#### 3.1.1 Doxorubicin

Doxorubicin is an anthracycline antibiotic commonly used to treat a variety of cancers, but the clinical use of doxorubicin is often limited by its cardiotoxicity (Singal and Iliskovic, 1998). The cardiotoxicity induced by anthracycline drugs may be a persistent phenomenon that begins with damage to cardiomyocytes; followed by left ventricular ejection fraction (LVEF), which, if not diagnosed and treated early, gradually leads to symptomatic heart failure (Cardinale et al., 2020), which may due to anthracycline drugs inhibit topoisomerase 2β, leading to mitochondrial dysfunction, activating cell death pathways and ROS deposition. Doxorubicin activates caspase-12, an apoptotic molecule that resides on the ER membranes, leading to cardiomyocyte apoptosis and cardiac insufficiency. Doxorubicin may induce UPR by down-regulating XBP1. Doxorubicin activates ER transmembrane stress sensors ATF6 in cultured cardiomyocytes and mouse hearts and suppresses the expression of XBP1. Reduced levels of XBP1 fail to induce expression of the ER chaperone glucose-regulated protein 78 (GRP78), which plays a major role in the UPR. Doxorubicin-triggered caspase-12 cleavage was well inhibited by heart-specific reversal of GRP78 downregulation, indicating that Doxorubicin causes deterioration of myocardial function through ATF6-XBP1-GRP78-Caspase-12 axis (Fu et al., 2016). Doxorubicin also prompts ER stress by regulating Gasdermin-D (GSDMD). RNA-seq showed that GSDMD activated ER-related genes to induce autophagy in the Doxorubicin-induced myocardial injury model. FAM134B is a common receptor involved in ER autophagy and mediates autophagosome recognition (Hetz et al., 2020; Mookherjee et al., 2021). Doxorubicin induces caspase-11 activation of GSDMD and increases the level of GSDMD in the ER. GSDMD promotes autophagy by promoting ER stress to activate FAM134B, thereby aggravating cardiomyocyte apoptosis (Qu et al., 2022). Beclin-1 is an important mediator of autophagosome formation and contains a BH3 domain that interacts with anti-apoptotic Bcl-2 family members. JNK activation upregulates Beclin-1 expression and phosphorylates Bcl-2, thereby promoting its dissociation from Beclin 1 and inducing autophagy (Kang et al., 2011). Since JNK is associated with ER stress through IRE1α activation, DOX-induced autophagy may be mediated by activation of IRE1α/JNK/Beclin-1 signaling (Yarmohammadi et al., 2021). Ca2+/calmodulin-dependent protein kinase II (CaMKII) is a multifunctional serine/threonine kinase that plays an important role in the regulation of intracellular calcium homeostasis and REDOX signaling, and mediates physiological and pathological responses to cardiac stress (Anderson, 2011; Mohler and Hund, 2011). Chronic and persistent CaMKII activation has detrimental effects on cardiac function and structure. Upon doxorubicin application, CaMKII exacerbates DOX-induced cardiotoxicity by promoting ER stress and apoptosis through the IRE1α/XBP1s pathway (Kong et al., 2022). Resveratrol (RV) is a phenol that has a variety of pharmacological effects. The protective effect of RV against Doxorubicin cardiotoxicity was reported to be mediated by inhibition of ER stress and activation of the Silent information regulator 1 (Sirt1) pathway. Sirt1 is known to downregulate ER stress-related genes and help maintain ER hemostasis (Liu et al., 2014). Sirt1 is a NAD-dependent class III histone deacetylase that plays a crucial role in cell survival (Brachmann et al., 1995). It negatively regulates the expression of pro-apoptotic proteins, such as caspase-3, a downstream activator of ER stress (Liu et al., 2014). In summary, ER stress may serve as an emerging target for the management of Doxorubicin-induced cardiotoxicity, and inhibiting DOX-induced ER stress may be a promising strategy for the treatment of DOX-related cardiotoxicity.

#### 3.1.2 Tyrosine kinase inhibitor

Tyrosine kinase inhibitors (TKIs) are widely used in clinical practice to treat cancers ranging from hematologic malignancies to advanced solid tumors. However, TKIs usually cause serious cardiac adverse events without a dose ceiling (Escudier et al., 2007; Llovet et al., 2008; Richards et al., 2011). Transcriptome analysis showed that three (afatinib, ponatinib, and sorafenib) of the eight widely used TKIs (afatinib, gefitinib, crizotinib, dasatinib, nilotinib, sorafenib, sunitinib, and ponatinib) induced ER stress in rat cardiomyocytes, causing cardiotoxicity. ER, stress-related genes (ATF4, NUPR1, DDIT3, TRIB3, CHAC1, SESN2 HERPUD1) were upregulated during this process. They may mediate ER stress-related cardiotoxicity by triggering the induction of different levels of lipid peroxidation, reactive oxygen species (ROS), calcium deficiency, and TNNT2 loss (Wang et al., 2023). The above study provides a new target for the treatment of cardiotoxicity in TKI therapy. However, the specific mechanisms of cardiotoxicity induced by TKIs through ER stress need to be further studied to guide clinical practice.

#### 3.1.3 Bevacizumab

Bevacizumab, a vascular endothelial growth factor signaling inhibitor, is a first-line immunotherapy agent for the treatment of lung cancer (Akamatsu et al., 2021). Bevacizumab-induced cardiotoxicity has been reported in recent years, including reduced left ventricular ejection fraction, hypertension, and arrhythmia (Van Leeuwen et al., 2020; Huang et al., 2021; Yetkin-Arik et al., 2021). Research showed that Bevacizumab reduced cardiomyocyte viability and function by inducing apoptosis. The study also found that Bevacizumab significantly increased the expression of CHOP and PERK in cardiomyocytes, demonstrated activation of ER stress, increased caspase-12 activity in a dose-dependent manner, and caused cardiomyocyte apoptosis (Li et al., 2021).

#### 3.1.4 Cisdiamine dichloroplatin

Cisdiamine dichloroplatin (Cisplatin) is a widely used chemotherapeutic drug, that can be used to treat a variety of entities including testicular tumors, ovarian germ cells, and cervical cancer (Osanto et al., 1992). The cardiac complications of cisplatin include various electrocardiographic changes, myocarditis, arrhythmia, congestive heart failure, and cardiomyopathy (Al-Majed et al., 2006). ROS formation is considered to be one of the causes of cardiotoxicity after cisplatin cancer chemotherapy, and it is also an important cause of ER stress (El-Awady et al., 2011). After cisplatin exposure, the expression levels of GRP78, phosphorylated PERK (p-PERK), calpain-1, caspase-12, CHOP, and p-eIF2α/total eIF2α ratio were increased in the heart, which induced ER stress-dependent and mitochondria-dependent apoptosis pathways. Blocking cisplatin-induced upregulation of GRP78, eIF2α, calpain-1, caspase-12, and CHOP by taurine could inhibit myocardial apoptosis, and improve prognosis (Chowdhury et al., 2016).

#### 3.1.5 5-Fluorouracil

5-Fluorouracil (5-FU) is an antitumor drug that mainly inhibits S-phase cells. 5-FU has a definite curative effect and strong anti-tumor effect and is the first-line option in the treatment of colorectal cancer, head and neck cancer, gastric cancer, pancreatic cancer, and breast cancer. However, 5-FU can cause progressive and irreversible cardiotoxicity, with an incidence of 1.2%–18.0% and associated mortality of 0%–8% (Sorrentino et al., 2012; Yuan et al., 2019). Recent studies have shown that 5-FU may induce ER stress in cardiomyocytes by up-regulating the ATF6 pathway, resulting in the increase of GRP78, CHOP, and PERK as well as ATF6 in myocardial tissues. Application of ATF6 inhibitor miRNA-199a-5p successfully attenuated 5-FU-induced cytotoxicity in cardiomyocytes, reduced LDH level, and increased SOD and GSH/GSSG ratio in 5-FU-treated rat heart tissues (Wang et al., 2024).

#### 3.1.6 Celastrol

Celastrol is a quinolomethyl triterpenoid with anti-cancer activity and is cardiotoxic. Celastrol administration induced cardiac insufficiency in mice in a dose-dependent manner, characterized by left ventricular dilatation, myocardial interstitial fibrosis, and cardiomyocyte hypertrophy (Liu et al., 2019). Celastrol induces ER stress and unfolded protein response, and induces CHOP-dependent pro-apoptotic effect in cardiomyocytes. siRNA targeting CHOP effectively prevented the pro-apoptotic effect of celastrol, indicating that the potential cardiotoxicity of serasterol is directly linked to ER stress (Chen et al., 2022).

### 3.2 Chaperone dysfunction

Constant chaperone surveillance is required to ensure protein homeostasis (Hartl et al., 2011). The molecular chaperone is defined as any protein that interacts stabilizes, or helps a non-native protein to acquire its native conformation (Hartl and Hayer-Hartl, 2009). Members of these protein families are commonly known as stress proteins or heat shock proteins (HSPs). Because under stress conditions, the concentration of protein folding intermediates increases and tends to aggregate, which correspondingly upregulates chaperones. There are five major categories of heat shock proteins: small heat shock proteins (sHsps), Hsp60, Hsp70, Hsp90, and Hsp100. Each class has multiple isoforms with their functions (Richter et al., 2010).

70-kDa heat shock proteins (Hsp70s) assist a wide range of folding processes, including the folding and assembly of newly synthesized proteins, refolding of misfolded and aggregated proteins, membrane translocation of organellar and secretory proteins, and control of the activity of regulatory proteins (Mayer and Bukau, 2005). ER-lumenal polypeptide chain binding protein Bip mentioned above is the most abundant member of the Hsp70 protein family in the ER lumen. Doxorubicin, as mentioned above, induces cardiotoxicity through the interaction between chaperone proteins and ER stress-related pathways. In particular, it was confirmed experimentally that Doxorubicin disrupted the expression of the Bip in murine hearts. Meanwhile, cardiac-specific overexpression of Bip chaperone can alleviate cardiac dysfunction induced by Doxorubicin (Fu et al., 2016). In addition, Hsp 70 directly interacts with Apaf-1 and inhibits apoptosome formation (Ravagnan et al., 2001). A previous study showed that rats treated with Doxorubicin showed increased apoptotic cardiomyocytes and Atorvastatin can inhibit this phenomenon and improve Doxorubicin-induced cardiac dysfunction through increasing Hsp70 expression (Gao et al., 2019; Li Y. et al., 2024).

Hsp90 has emerged as a promising therapeutic strategy for the treatment of cancer, as many of the HSP90 client proteins are intimately involved in oncogenic progression (Bishop et al., 2007). However, some toxicological detriments have arisen, such as cardiotoxicity resulting from ether-a-go-go-related gene (hERG) inhibition following the administration of Hsp90 inhibitors (Biamonte et al., 2010). hERG encodes the α-subunit of the voltage-gated potassium channel, constituting a major component of the ion channel responsible for the repolarization of the cardiac action potential (Vandenberg et al., 2012). hERG has been previously confirmed as a client protein that depends upon Hsp90 for its folding and maturation. Geldanamycin, a specific Hsp90 inhibitor, causes misfolding and increases proteasomal degradation of hERG, which can cause long QT syndrome, resulting in ventricular arrhythmias and even death (Vandenberg et al., 2001; Ficker et al., 2003). Meanwhile, hERG depends upon a single Hsp90 isoform, and the genetic knockdown of Hsp90α, but not Hsp90β, resulted in a trafficking-defective hERG channel (Peterson et al., 2012).

### 3.3 Impairment of the proteasome system

In eukaryotic cells, the ubiquitin-proteasome system (UPS) is the principal pathway for the targeted degradation of mutated, oxidized, or misfolded proteins, which plays a significant role in the maintenance of proteostasis (Pokorna et al., 2019). For the most part, the ubiquitin system labels the target protein for recognition by the 26S proteasome, formed by the 20S proteasome core complex and two flanking 19S cap structures. A small fraction of proteins such as oxidized proteins are recognized directly by the 20S proteasome complex (Herrmann et al., 2013). In cardiomyocytes, various forms of cardiac stress, such as pressure overload and oxidative damage, can lead to the misfolding of proteins, which are then targeted for destruction by the UPS to maintain cell integrity (Tannous et al., 2008). The inhibition of proteasomal-dependent protein turnover can lead to the abnormal accumulation of misfolded proteins that associate with one another to form sequentially higher-order protein aggregates toxic to cellular function (Wu et al., 2020). These abnormal ubiquitinated protein aggregates including soluble oligomers and aggresomes can cause cellular injury and ultimately lead to caspase-mediated apoptosis and cell death which have been found in human cardiomyopathies including hypertrophic cardiomyopathy (HCM), dilated cardiomyopathy (DCM) as well as heart failure (HF) (Predmore et al., 2010; Barac et al., 2017; Hasinoff et al., 2017). Additionally, proteasome inhibition can lead to the accumulation of mutant proteins and the generation of amyloid oligomers and cardiac, and intrasarcoplasmic amyloidosis, contributing to the stiffening of the ventricle and a restrictive filling pattern (Herrmann et al., 2013).

Multiple myeloma is a hematological cancer characterized by an abnormal proliferation of clonal plasma cells. The treatment of multiple myeloma has made significant strides in recent years due to a deeper understanding of the illness and the accelerated development of new medications (Zheng et al., 2023). Bortezomib, carfilzomib, and ixazomib are three proteasome inhibitors approved for use in the treatment of multiple myeloma (Rajkumar, 2020). However, the use of proteasome inhibitors is associated with a variety of cardiovascular complications, including hypertension, pulmonary hypertension, heart failure, arrhythmias, ischaemic heart diseases, and thromboembolism (Zheng et al., 2023). In myeloma cells, proteasome inhibition results in the rapid accumulation of misfolded protein and leads to ER stress which culminates in UPR with induction of apoptotic cascade (Obeng et al., 2006). Cardiomyocytes also exhibit particular vulnerability to proteasome inhibition, given their non-proliferative state and elevated proteasome activity in comparison to other tissues (Patel and Majetschak, 2007). Therefore, carfilzomib, an irreversible proteasome inhibitor, is the most strongly associated with cardiotoxicity which may involve the ubiquitin-proteasome system (Zheng et al., 2023). Approximately 80% of carfilzomib-treated patients experienced proteasome inhibition, and prolonged inhibition of the ubiquitin-proteasome system results in the accumulation of misfolded proteins in cardiomyocytes, leading to adverse cardiac remodeling, as observed in dilated and hypertrophic cardiomyopathies (Pagan et al., 2013; Grandin et al., 2015). Interestingly, bortezomib, a reversible proteasome inhibitor that has been in clinical use for more than a decade, has not been associated with significant cardiac toxicity. It appears that in some cases, a low dose of bortezomib prevents hypertrophy and reverts myocardial remodeling (Meiners et al., 2008). In comparison to bortezomib, carfilzomib binds irreversibly rather than slowly reversibly to the 20S proteasome β-subunit and more specifically targets the chymotrypsin catalytic subunit which suggests that the specific effects of proteasome inhibition on cardiac structure and function may depend on its dose, magnitude, duration and timing about other cardiac stressors (Grandin et al., 2015).

### 3.4 Dysregulated autophagy

Autophagy is a highly conserved biological process and its main function is to maintain cellular homeostasis after stress stimuli, such as nutrient starvation, energy depletion, and impairment of the redox state, promoting the removal of damaged or long-lived organelles, misfolded or aggregated proteins, and intracellular pathogens (Chun and Kim, 2018). The activation of autophagy has been proven to protect the heart from different types of stress, including protein misfolding-induced cardiomyopathy (Bhuiyan et al., 2013). In contrast, impairment of cardiac autophagy can amplify unfolded or misfolded proteotoxicity and result in cardiomyopathy (Zaglia et al., 2014).

Anthracyclines (AC) induced cardiotoxicity was initially attributed to inducing cell-damaging oxidative stress, while antioxidants failed to prevent anthracycline-induced cardiotoxicity, suggesting that additional mechanisms are involved, among which autophagy was shown to play an important role (Ghigo et al., 2016; Varricchi et al., 2018a). The initiation of the autophagic process is under the control of unc-51-like autophagy activating kinase 1 (Ulk-1) complex and the kinase activity of Ulk-1 is mediated by AMP-activated protein kinase (AMPK) and mammalian target of Rapamycin (mTOR) (Kim et al., 2011; Nazarko and Zhong, 2013). Anthracyclines can block autophagy initiation by controlling mTORC1 and AMPK and reactivating autophagy can protect the heart against anthracycline-induced cardiotoxicity (Li et al., 2018). However, some studies have shown that activation of autophagy is a primary cause of cardiomyocyte programmed cell death in response to AC and the conflicting findings on the role of autophagy in AC-induced cardiotoxicity may stem from the lack of a systematic and accurate analysis of the autophagic flux as well as from the different dose of AC (acute high-dose vs. chronic low-dose), potentially affecting the process of autophagy at different steps and extents (Li et al., 2020).

## 4 Targeting proteostasis to prevent drug-induced cardiotoxicity

In comparison to other cell types, cardiomyocytes are of particular necessity to regulate proteostasis and are therefore susceptible to the side-effects of drugs due to their highly specific functions (electrical conduction, contraction), high metabolic demands, and terminally differentiated state (Henning and Brundel, 2017). At present, three categories of primary prevention measures for DICT are in use (Curigliano et al., 2016). The most common is dose restriction, but this is likely to affect antitumor therapy outcomes. Furthermore, there is no absolute safe dose for a particular drug due to individual differences. The second category comprises the use of alternative treatments or changes in the mode of administration. The third category is the combination of cardioprotective drugs, such as atorvastatin and sacubitril/valsartan (Li et al., 2022), but their actual utility has yet to be verified in further basic and clinical trials.

The only currently approved pharmaceutical agent for the prevention of anthracycline-induced cardiotoxicity is dexrazoxane (Langer, 2014). However, the scenarios for its use are limited, thereby necessitating the development of novel drug therapies. The mechanism by which the aforementioned pharmaceutical agents induce cardiotoxicity by disrupting proteostasis presents a promising avenue for mitigating DICT. Geranylgeranylacetone (GGA) is the most intensively studied of the compounds under consideration. It is not only a widely used antiulcer drug but also a non-toxic inducer of HSP expression. It has been demonstrated to rapidly increase the expression of HSPs in response to ischemia, hypoxia, oxidative stress, and toxicity, resulting in a significant protective effect. Its efficacy has been established in the prevention and treatment of myocardial ischemia-reperfusion injury and atrial fibrillation (Zeng et al., 2018). Notably, in doxorubicin-induced cardiotoxicity, GGA could inhibit oxidative stress and apoptosis in cardiomyocytes by upregulating HSP90 (Fujimura et al., 2012; Sysa-Shah et al., 2014). Similarly, Salubrinal upregulated the chaperone levels in cardiomyocytes via suppressing the dephosphorylation of eIF2A, thus protecting against ER-stress induced apoptosis (Li et al., 2015). Furthermore, a recently published study has confirmed a novel mechanism by which low-dose colchicine attenuates doxorubicin-induced cardiotoxicity by promoting autolysosome degradation through microtubule regulation (Peng et al., 2024). Given its low cost, long-term safety, and good tolerability, colchicine has the potential to be an optimally suited drug candidate. The subsequent phase of research should concentrate on the cardiac-specific regulation of proteostasis, to develop innovative drug therapies that can mitigate cardiotoxicity without affecting the efficacy of the chemotherapeutic agents themselves.

## 5 Monitoring proteostasis to assess potential cardiotoxicity

The prevailing methodologies for evaluating drug cardiotoxicity concentrate on cumulative dose exposure to drugs and the monitoring of cardiac function (ultrasound, electrocardiogram, etc.), yet they are deficient in terms of temporal precision and specificity. Technological advances and methodological breakthroughs in recent years have enhanced our comprehension of protein misfolding. The monitoring of protein homeostasis in clinical samples may prove to be a promising avenue for effectively assessing potential drug cardiotoxicity. Furthermore, advancements in preclinical trial modeling are occurring simultaneously with the monitoring of protein homeostasis (Lee et al., 2024). Among them, human induced pluripotent stem cell-derived cardiomyocytes (iPSC-CMs) are a highly effective platform (Burnett et al., 2021). Adult CMs and iPSC-CMs exhibit a significant degree of similarity in terms of their electrophysiology, cellular signaling, contractile processes, and metabolism (Lindenstrauß et al., 2017). The intricate and coordinated functions have direct relevance to the assessment of toxicity.

As mentioned previously, several spectroscopic methods have been used to directly monitor the structural changes and protein conformation. Our current priority is to explore the practical application of these approaches in the *in vivo* setting (Doerr, 2014). LiP-MS, or Limited Protein Hydrolysis Coupled Mass Spectrometry, is a newly developed proteomics technique that can detect alterations in protein structure within intricate biological settings, covering the entire proteome (Cappelletti et al., 2021). The quantification of conformationally specific peptides produced by protease digestion allows for the study of the structure of disease-related proteins in clinical samples, enabling the selection of potential biomarkers. LiP-MS screening of proteomic changes in cerebrospinal fluid (CSF) identified structural changes in proteins in the CSF that are closely associated with neurodegenerative pathologies (Shuken et al., 2022). Furthermore, LiP-MS analysis of samples in the presence of drug candidates permits a direct assessment of the effect of drug candidates on aggregation processes (Holfeld et al., 2024). Further, Currie et al. developed an experimental strategy and computational analysis workflow to perform simultaneous proteome localization and turnover (SPLAT) measurements (Currie et al., 2024). The spatiotemporal proteomic data obtained from carfilzomib-intervened IPSC-CM revealed significant disturbances in proteasome remodeling, the induction of chaperone and ERAD proteins, and the mitochondrial protein quality control mechanisms. Additionally, a preferential decrease in sarcomere indicates that it may be the primary site of lesion for carfilzomib cardiotoxicity. Furthermore, differential protein abundance analysis revealed significant upregulation of MHC-β (MYH7) and desmoplakin (DSP), suggesting that similar protein deposition lesions may underlie the mechanism of cardiotoxicity *in vivo*.

In addition, thermal proteome profiling (TPP), which combines cellular thermal shift assay (CETSA) and quantitative mass spectrometry (MS), can depict the melting profile of thousands of proteins. With the addition of a specific drug, the melting curves are evaluated for differences, thus the target proteins were identified (Saei et al., 2020). Perrin et al. exploited tissue-TPP and blood-TPP techniques to reveal the proteome-wide heat stability profiles of the deacetylase inhibitor panobinostat, as well as the B-Raf inhibitor vemurafenib, and to derive their target profiles (Perrin et al., 2020). These approaches will help to elucidate the mechanism of drug action *in vivo* and make systematic assessments of the stability of target proteins.

Given that these proteostructural monitoring methods have only recently emerged, the related basic experiments and clinical studies are still in their infancy. Fortunately, preliminary basic studies have demonstrated the unique value of these methods in elucidating protein homeostasis, including target identification, spatial localization, and quantitative analysis. We anticipate that the relevant technologies will prove invaluable in the subsequent development of new drugs or monitoring of drug efficacy.

## 6 Conclusion

The research about the dynamics of proteostasis is going through a paradigm shift. Increasingly more people are emphasizing the need to establish an entire *in vivo* research system. Relevant research should concentrate on the dynamic monitoring of proteostasis and its control systems, particularly chaperones, proteasomes and others. It can be concluded that antineoplastic drugs have the potential to disrupt cardiac proteostasis through four mechanisms, including the induction of ER stress and UPR, the interference with the expression or function of chaperones, the alteration of proteasome function, and the influence of autophagy, which can result in cardiotoxicity such as cardiomyopathy, myocarditis, and arrhythmia. This represents a novel avenue for the prevention and treatment of DICT, whereby pharmacological agents targeting proteostasis, including GGA, can be employed to mitigate proteotoxicity. The advent of novel *in vivo* protein monitoring techniques, such as LiP-MS, has the potential to furnish more accurate insights into proteostasis. What’s more, they also offer a novel approach to future drug development, whereby *in vivo* model-based high-throughput screening techniques are employed to identify pharmacological targets, thus mitigating the risk of cardiotoxicity.
